# Decreased Intrathecal Concentrations of Free Light Chains Kappa in Multiple Sclerosis Patients Taking Very High Effective Disease-Modifying Treatment

**DOI:** 10.3390/diagnostics12030720

**Published:** 2022-03-16

**Authors:** Marie Süße, Franz Felix Konen, Philipp Schwenkenbecher, Kathrin Budde, Matthias Nauck, Matthias Grothe, Malte Johannes Hannich, Thomas Skripuletz

**Affiliations:** 1 Department of Neurology, University Medicine Greifswald, 17475 Greifswald, Germany; matthias.grothe@med.uni-greifswald.de; 2 Department of Neurology, Hannover Medical School, 30625 Hannover, Germany; konen.felix@mh-hannover.de (F.F.K.); schwenkenbecher.philipp@mh-hannover.de (P.S.); skripuletz.thomas@mh-hannover.de (T.S.); 3 Institute of Clinical Chemistry and Laboratory Medicine, University Medicine Greifswald, 17475 Greifswald, Germany; kathrin.budde@med.uni-greifswald.de (K.B.); matthias.nauck@med.uni-greifswald.de (M.N.); malte.hannich@med.uni-greifswald.de (M.J.H.)

**Keywords:** cerebrospinal fluid, immunoglobulin synthesis, free light chain kappa, oligoclonal IgG, multiple sclerosis, disease-modifying therapy

## Abstract

Free light chains kappa (FLCκ) in cerebrospinal fluid (CSF) are a part of the intrathecal immune response. This observational study was conducted to investigate the effects of different disease-modifying therapies (DMT) on the humoral intrathecal immune response in the CSF of patients with multiple sclerosis (MS). FLCκ were analyzed in CSF and serum samples from MS patients taking DMT (*n* = 60) and those in a control cohort of treatment-naïve MS patients (*n* = 90). DMT was classified as moderately effective (including INFß-1a, INFß-1b, glatiramer acetate, dimethyl fumarate, teriflunomide, triamcinolone); highly effective (including fingolimod, daclizumab) and very highly effective (alemtuzumab, natalizumab, rituximab/ocrelizumab, mitoxantrone). FLCκ were measured using a nephelometric FLCκ kit. Intrathecal FLCκ and IgG concentrations were assessed in relation to the hyperbolic reference range in quotient diagrams. Intrathecal FLCκ concentrations and IgG concentrations were significantly lower in samples from the cohort of MS patients taking very highly effective DMT than in samples from the cohort of MS patients taking highly effective DMT and in the treatment-naïve cohort (FLCκ: *p* = 0.004, *p* < 0.0001 respectively/IgG: *p* = 0.013; *p* = 0.021). The reduction in FLCκ could contribute to an anti-inflammatory effect in the CNS through this mechanism. There was no difference in the appearance of CSF-specific oligoclonal bands (*p* = 0.830). Longitudinal analyses are required to confirm these results.

## 1. Introduction

A pathophysiological important component in the development of inflammatory activity in multiple sclerosis (MS) is intrathecal immunoglobulin (Ig), which is produced by plasma blasts and plasma cells [1,2,3]. During the production and secretion of Ig, free light chains are released, which thus also represent a cerebrospinal fluid (CSF) biomarker for inflammatory activity [4,5,6]. In particular, the free light chains of the kappa isoform (FLCκ) allow reliable and rapid analysis by assays that are now commercially available. They are at least equivalent to the parameters previously used in clinical routine in terms of diagnostic sensitivity with regard to intrathecal inflammation [4,7,8]. The FLCκ index and the interpretation of FLCκ in quotient diagrams have been investigated in numerous studies for their value in the diagnosis of MS [1,7,8,9,10]. Nevertheless, little is known about the changes of FLCκ concentrations due to disease-modifying therapies (DMT) in MS, which may be due to the rarity of CSF analyses during the course of MS disease. Depending on the therapeutic target, moderately, highly, and very highly effective therapeutics are available based on the reduction in relapse rates in pivotal trials [11,12]. The occurrence of CSF-specific oligoclonal bands (OCB) as the current standard for the detection of intrathecal inflammation hardly changes after the use of drugs, such as rituximab or natalizumab [2,13,14,15,16]. Since FLCκ represents products of Ig synthesized by B cells, they could serve as biomarkers for B cell depleting therapies [17]. As the exact role of B cells and the intrathecal humoral immune response in the pathophysiology of MS remains unclear and a better understanding of their role is needed to refine therapeutic approaches [3], changes in the CSF biomarker profile may provide important information on this topic. The aim of this study was to investigate the effects of different DMT on the intrathecal humoral immune response, as reflected by intrathecal FLCκ and IgG concentrations. 

## 2. Materials and Methods

This two-center study evaluates data from patients treated at the Department of Neurology, Hannover Medical School (MHH) between 2010 and 2021 or the Department of Neurology, University Medicine Greifswald (UMG) between 2008 and 2019. Primary selection criteria were patients with a confirmed diagnosis of MS according to current diagnostic criteria [18,19,20] and CSF analysis while taking DMT, and available stored paired CSF and serum samples. In addition, MS patients not taking DMT at the time of CSF analysis were identified as a control cohort. Paired CSF and serum samples were collected as part of routine diagnostic procedures. Clinical data were collected by reviewing the patients’ medical records. Some of these patient samples had been previously investigated under different aspects [1,7,21,22,23]. 

All samples were analyzed according to routine diagnostics in the Neurochemistry Laboratory of the Department of Neurology of the MHH and the Interdisciplinary CSF Laboratory of the UMG. Kinetic nephelometry (Beckman Coulter IMMAGE, Brea, CA, USA (at the MHH)); BN ProSpec, (Siemens Healthcare Diagnostics Products GmbH, Marburg, Germany (at the UMG)) were used to measure concentrations of albumin, IgG, IgM, and IgA in serum and CSF samples. Oligoclonal bands were detected by isoelectric focusing in polyacrylamide gels, followed by silver staining (MHH) or using isoelectric focusing with a semiautomatic agarose electrophoresis system (Hydragel 9 CSF, Hydrasys 2Scan, Sebia GmbH, Fulda, Germany) (UMG). Free light chains kappa in sera and CSF were measured by nephelometry using the N Latex FLC kappa kit (Siemens Healthcare Diagnostics Products GmbH, Marburg, Germany) according to the manufacturer’s protocol on the BN ProSpec analyzer at both sites. The pre-dilution of CSF was set to 1:2, the pre-dilution of the serum to 1:100. The lower limit of quantification of the assay was 0.034 mg/L. 

To calculate the amount of intrathecally synthesized FLCκ (FLCκ loc) and the amount of intrathecally synthesized IgG (IgG loc), we used the formula suggested by Reiber et al. [8]:

FLCκ loc = [Q FLCκ (total) − Q FLCκ (mean)] × FLCκ serum [mg/L](1)

IgG loc = [Q IgG (total) − Q IgG (mean)] × IgG serum [mg/L](2)

### Statistical Analysis

SPSS 25.0 (IBM Co., Armonk, NY, USA) and RStudio (R version 3.5.1 2 July 2018) were used for the statistical and graphical processing of the data. Kolmogorov Smirnov analysis was used to test for a Gaussian distribution of the data. Statistical significance was assessed using the chi-square test for nominal data. A comparison between groups was performed using the Mann–Whitney U test or the Kruskal–Wallis test (no Gaussian distribution of data). The *p* values ≤ 0.05 were considered statistically significant. The Dunn–Bonferroni test was used for post hoc analysis.

## 3. Results

### 3.1. Patients’ Characteristics

Basic clinical and CSF data for the cohorts are shown in Table 1. Initially, 66 patient samples were identified that met the primary selection criteria for patients taking DMT. Of these, 6 patient samples with serum FLCκ values above the upper limit range (according to manufacturer’s specification: 6.7–22.4 mg/L) were excluded, since these may cause a falsely low FLCκ intrathecal fraction (IF) or index [4,24]. In total, the laboratory results of 60 paired serum and CSF samples from 56 patients were included in the further analyses. These patient samples were grouped into cohorts according to the efficacy of the respective DMT [11,12]: (cohort I) moderate efficacy: INFß-1a (*n* = 5), INFß-1b (*n* = 3), glatiramer acetate (*n* = 2), dimethyl fumarate (*n* = 4), teriflunomide (*n* = 3), triamcinolone (*n* = 3); (cohort II) high efficacy: fingolimod (*n* = 7), daclizumab (*n* = 3); (cohort III) very high efficacy: alemtuzumab (*n* = 8), natalizumab (*n* = 14), rituximab/ocrelizumab (*n* = 7), mitoxantrone (*n* = 1). Intrathecal triamcinolone acetonide (TCA), a synthetic steroid, is not regarded as a disease-modifying therapy per se, and its use in MS is controversial. This will be addressed later in the discussion. Daclizumab was used, as MS DMT and has since been withdrawn from the market due to safety concerns. The reasons for CSF analysis were: intrathecal use of TCA (*n* = 3), exclusion of progressive multifocal leukoencephalopathy (PML) (*n* = 12), exclusion of other CNS infections (*n* = 45). Patient samples obtained from diagnostic CSF analysis at the time of MS diagnosis without DMT (*n* = 90) were selected as the control group. 

Patients under treatment with a moderately effective DMT were significantly older than patients taking very highly effective DMT and patients without DMT (*p* = 0.025, *p* = 0.002, respectively). Patients in cohort I had a lower number of previous DMT than patients in cohorts II and III (*p* < 0.0001). No significant difference was found between the three cohorts with DMT in their disease duration or disease stage, assessed by the expanded disability status scale (EDSS) (*p* = 0.374, *p* = 0.113, respectively). Patients without DMT had significantly lower EDSS scores than patients on highly and very highly effective DMT (*p* = 0.003, *p* < 0.0001, respectively). Patients on moderately effective DMT were more likely to meet the NEDA-3 criteria [25] than patients taking very high or high effective DMT (Table 1).

### 3.2. Impact of DMT on Intrathecal IgG Concentrations and OCB Status

**Figure 1:**
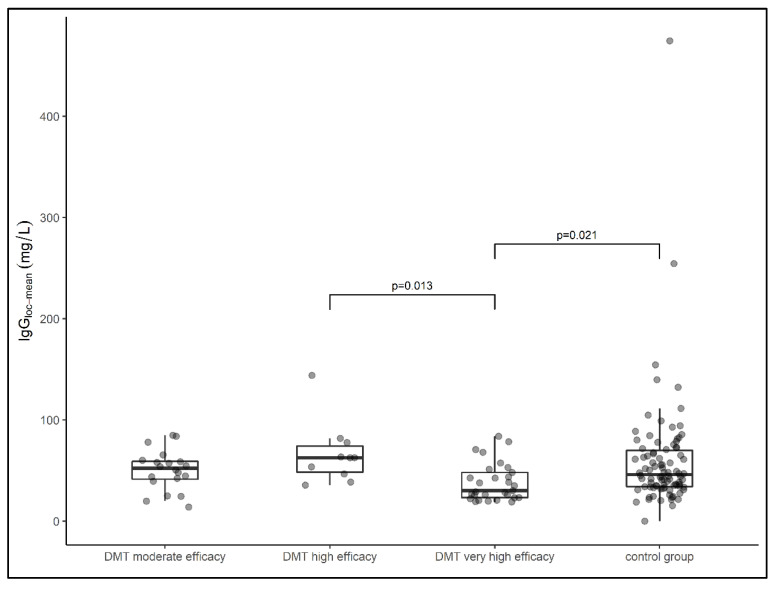
Data on local IgG concentrations of patient samples presented in box plots (median, first, and third quartile). Samples from patients treated with very highly effective DMT had significantly lower IgG concentrations than samples treated with highly effective DMT (*p* = 0.013) and the control group (*p* = 0.021). DMT—disease-modifying therapy, Ig—Immunoglobulin. Comparison between groups was performed using the Kruskal–Wallis test. The Dunn–Bonferroni test was used for post hoc analysis.

The median local IgG concentration relative to the Qmean was highest in the group of samples from patients treated with highly effective DMT (median IgG loc (mean) = 62.7 mg/L), followed by samples from patients treated with moderate effective DMT (median IgG loc (mean) = 52.3 mg/L) and samples from patients in the treatment-naïve group (median IgG loc (mean) = 46 mg/L). The lowest local IgG concentration was found in samples from patients treated with very high effective DMT (median IgG (mean) = 34 mg/L). The latter group had a significantly lower IgG concentration than the samples treated with highly effective DMT (*p* = 0.013) and samples from patients in the control group (*p* = 0.021) (Figure 1). When comparing all DMT cohorts with the treatment-naïve cohort, there was no significant difference in local IgG concentrations (*p* = 0.207).

With regard to the individual drug classes, samples from patients treated with fingolimod had the highest local IgG concentrations, followed by dimethyl fumarate, interferon, mitoxantrone, triamcinolone, teriflunomide, daclizumab, natalizumab, glatiramer acetate, anti-CD20 therapies, and alemtuzumab (Figure 3). No difference in the prevalence of CSF-specific OCB was demonstrated between the four cohorts (*p* = 0.826).

### 3.3. Impact of DMT on Intrathecal FLCκ Concentrations

**Figure 2:**
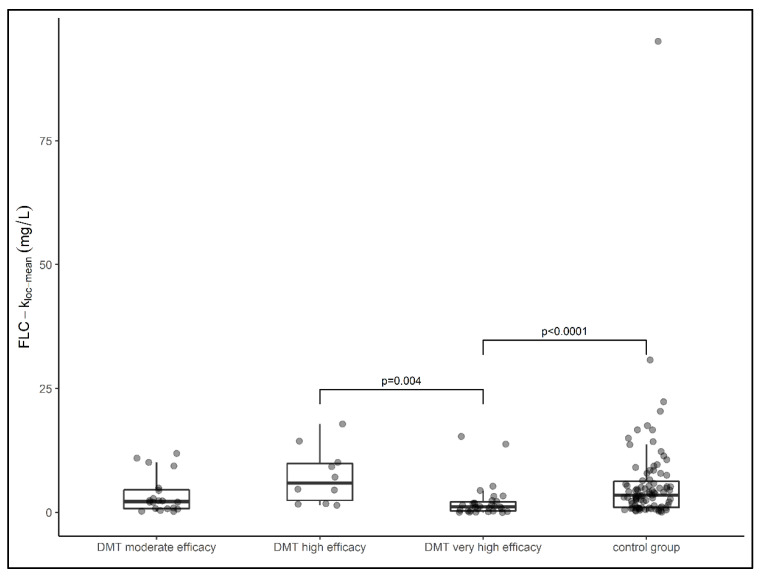
Data of local FLCκ concentrations of patient samples presented in box plots (median, first, and third quartile). Samples from patients treated with very high effective DMT had significantly lower FLCκ concentrations than samples treated with high effective DMT (*p* = 0.004) and the control group (*p* < 0.0001). DMT—disease-modifying therapy, FLCκ—free light chains kappa. Comparison between groups was performed using the Kruskal–Wallis test. The Dunn–Bonferroni test was used for post hoc analysis.

**Figure 3:**
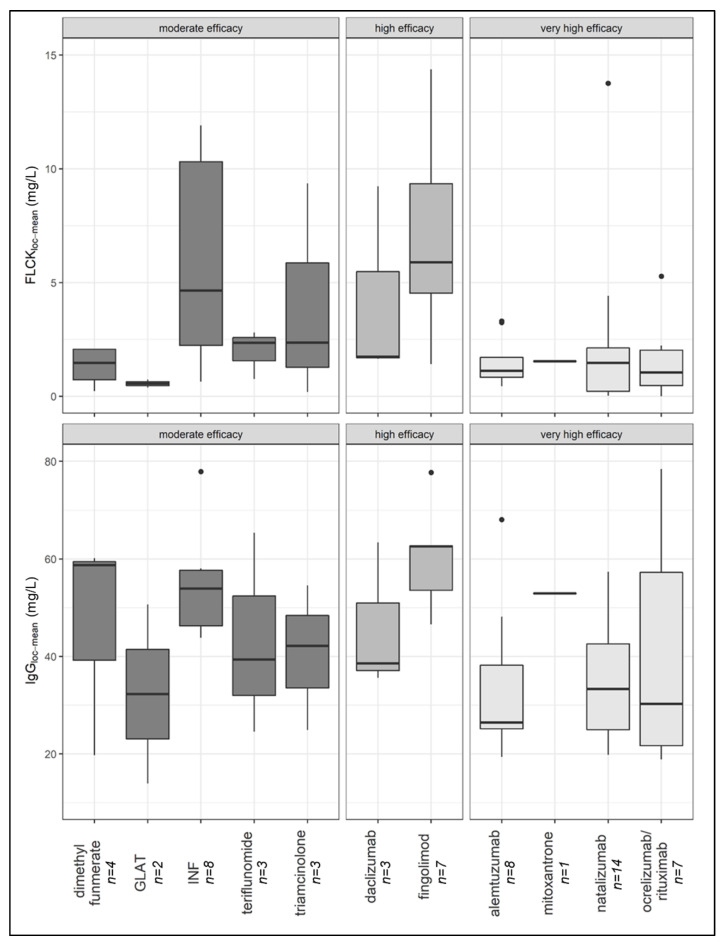
Local FLCκ and IgG concentration of patient samples distributed after ingestion of DMT (median, first, and third quartile)**.** FLCκ—free light chains kappa, DMT—disease-modifying therapy, INF—interferon, GLAT—glatiramer acetate.

The median local FLCκ concentration relative to the Qmean was highest in the group of samples from patients treated with highly effective DMT (median FLCκ loc (mean) = 5.89 mg/L), followed by samples from patients in the treatment-naïve cohort (median FLCκ loc (mean) = 3.49 mg/L) and samples from patients treated with moderately effective DMT (median FLCκ loc (mean) = 2.19 mg/L). The lowest local concentration of FLCκ was found in samples from patients treated with very highly effective DMT (median FLCκ loc (mean) = 1.12 mg/L). The latter group had a significantly lower FLCκ concentration than samples from patients treated with highly effective DMT (*p* = 0.004) and samples from patients in the control group (*p* < 0.0001) (Figure 2). Comparing all DMT cohorts with the treatment-naïve cohort, the local FLCκ concentrations were significantly lower in patients taking DMT (*p* = 0.007). With regard to the individual drug classes, samples from patients treated with fingolimod had the highest local FLCκ concentrations, followed by interferon, triamcinolone, teriflunomide, daclizumab, mitoxantrone, dimethyl fumarate, alemtuzumab, anti-CD20 therapies, natalizumab, and glatiramer acetate (Figure 3).

## 4. Discussion

Although the mechanism of the intrathecal humoral immune response in MS is not yet fully understood, it is known to play an important role in disease pathogenesis [2,13]. Some aspects have already been investigated, such as the prognostic value of intrathecal IgM synthesis or the relationship between intrathecal IgG synthesis and clinical disease progression [26,27].

This study was conducted to investigate the effect of DMT on the intrathecal humoral immune response as reflected by intrathecal FLCκ and IgG concentrations. There is very little data on the humoral immune response in the CSF of MS patients undergoing DMT. FLCκ are a quantifiable and very sensitive promising biomarker for intrathecal inflammation [8]. The question is whether they can better reflect the efficacy of modern MS therapeutics than other biomarkers of intrathecal inflammation. OCB, as the previous reference standard for intrathecal IgG synthesis, is limited to a qualitative interpretation and is not suitable as a progression parameter for changes in the humoral immune response of the CNS.

The presence of CSF-specific OCB does not change during the disease course of MS. A possible explanation for this is their production by long-lived plasma cells that migrate into a survival niche in the CNS [2]. Our results are consistent with this, as the frequency of CSF-specific OCB in our study was not influenced by the intake of different DMTs.

The most important finding of our study was the observation that in samples from patients on very high effective DMT, local FLCκ, and IgG concentrations were significantly lower compared to untreated and other DMT classes.

Some of these very effective approved therapies for MS directly target B cells [3]. Since intrathecal B cells are the origin of FLCκ [3,5], our study finding is not surprising in itself. The accumulation of B cells causes or contributes to a worse clinical course in MS patients [3]. B cells release inflammatory and cytotoxic mediators into the CSF, creating an intracerebral milieu that perpetuates chronic compartmentalized inflammation and also directly mediates or exacerbates cortical pathology and disease progression [3,28]. Ocrelizumab, ofatumumab, or rituximab, as B cell therapies, have been tested in clinical trials to date. However, there are no data on the effect of ocrelizumab on the humoral intrathecal immune response and very little data on the effect of other CD20 depleting therapies. Rituximab administered intrathecally did not alter the IgG index, IgG concentration, or OCB band counts in one patient who received repeated CSF analyses [29]. Even though the number of B cells in the CNS was significantly reduced by intravenous therapy with rituximab, the IgG concentrations, IgG index, and CSF-specific OCB remained detectable [15]. The authors suggested that the effect of B cell depletion on central inflammatory markers and neurodegeneration is independent of antibody-mediated responses and that the majority of Ig is secreted by plasma cells that do not express CD20 [15,29,30]. In line with these considerations, minimal changes in absolute FLCκ concentrations in CSF were reported in a clinical trial investigating intrathecally applied rituximab [31]. On the other hand, long-term humoral immunity is thought to be due to periodic non-specific activation of CD20 expressing memory B cells [30], which may help explain our divergent study results. In addition, it must be taken into account that the cohort of very high effective DMT also included patients who received DMTs that do not directly or exclusively target B cells, such as natalizumab and alemtuzumab. However, most of the knowledge of treatment associated with biomarker changes in CSF for inflammation in MS patients is on natalizumab, as this therapy has a particularly high risk of PML, which can be confirmed by CSF analysis [2,16,32,33]. Natalizumab prevents immune cells from migrating across the blood-brain barrier by targeting α4-integrin, thereby reducing the number of CSF TCD4+, TCD8+, BCD19+, and plasma CD138+ cells [2]. As a consequence, patients treated with natalizumab have a lower proportion of B cells in the CSF compared to untreated MS patients [32,33]. IgG and IgM concentrations, IgG index, and intrathecal IgG fraction decreased in longitudinal samples during natalizumab therapy, while OCB in CSF remained in most cases [2,19,32,33]. Only 16–18% lost OCB in CSF during natalizumab therapy [2,33]. In contrast, other authors reported a higher frequency of OCB disappearance in up to 67% after only the second infusion with natalizumab [13]. The reason for these findings is seen in the fact that natalizumab reduces short-lived plasma blasts in the CNS compartment but has little effect on locally persistent long-lived plasma cells [2]. The restricted number of B lymphocytes and the impaired humoral immune response under natalizumab therapy are thought to be responsible for the increased risk of PML [16,32]. There is limited data on intrathecal IgG concentrations in samples from patients treated with alemtuzumab, a humanized monoclonal antibody that selectively binds CD52 expressed on the surface of T and B lymphocytes [34]. A recently published study demonstrated a significant decrease in the intrathecal IgG fraction [35]. The authors concluded that a decrease in intrathecal IgG concentration could suppress the autoimmune process in the CNS [34]. In contrast to these study results, no reduction in the FLCκ index was observed after 24 months of treatment with alemtuzumab [35].

In the highly effective DMT cohort, most patients in our study received fingolimod. Fingolimod acts as a superagonist for the sphingosine-1-phosphate receptor 1 and traps lymphocytes in secondary lymphoid organs, reducing migration of these cells into the CNS [36]. Fingolimod has little effect on the intrathecal humoral immune response, as reflected by unchanged intrathecal IgG concentrations, IgG index, FLCκ index, and sustained OCB at 75% before and during treatment [35,36]. While a general decrease in CSF leukocytes during therapy has been described [36], the fraction of CSF B cells was increased in fingolimod-treated patients with MS [36]. This observation could also explain the higher median FLCκ concentration in samples from fingolimod-treated patients compared to the other treatment groups and the treatment-naïve cohort of patients in our study. The cohort of patients taking highly effective DMT not only had the highest local FLCκ concentrations but also had a more frequent Gd uptake on MRI and EDSS progression before an LP, and thus, were less likely to reach the NEDA-3 criteria. Thus, the high FLCκ levels in this cohort may reflect increased disease activity, although a generalization is not recommended due to the small cohort. However, the fact that the local FLCκ levels are lower in patients receiving very highly effective DMT contradicts this hypothesis, even though the NEDA rate is also very low at 16.7%. However, our results confirm the statements of a recent publication in which no intraindividual decrease in the FLCκ index could be observed when taking fingolimod [35].

Other DMT, such as dimethyl fumarate, teriflunomide, IFNβ, or glatiramer acetate, can also affect B cells and thus the intrathecal humoral immune response, even if they are not the primary target. For example, INFß-1b therapy reduces the number of circulating CD 80+ B cells [37]. In addition, treatment with INFß-1a resulted in the normalization of initially elevated CSF cell counts and CSF FLCκ concentrations in approximately 25% and 15% of patients, respectively [14]. This could be an explanation for the lower local FLCκ concentrations in this DMT group compared to the treatment-naïve patient cohort in our study.

Intrathecal triamcinolone acetonide (TCA), a synthetic steroid, is not a disease-modifying therapy per se and its use in MS is controversial. Intrathecal TCA is a therapeutic option for MS patients with predominantly spinal symptoms, such as spasticity. In addition to its antispastic effect, it is also believed to have regenerative effects [38]. Thus far, there is no data on possible changes in the humoral immune response by TCA.

However, the central question concerns the relevance of reduced intrathecal IgG and FLCκ concentrations in response to very high effective DMT [33]. The biological function of FLCκ as part of the intrathecal humoral immune response is still unclear. It has been suggested that FLC themselves have several biological functions. These include their ability to modulate the immune system. The activation of the complement cascade, proteolytic activity, and the ability to bind to antigenic structures and chemotactic factors are examples [39]. They are able to sensitize mast cells, leading to mast cell degranulation and de novo synthesis and a release of inflammatory mediators [39]. Interestingly, an increase in specific mast cell markers can be seen in MS patients in combination with increased FLC dimers [39,40]. One could speculate that the reduction in FLCκ concentrations by very highly effective DMT might also reduce FLCκ’s own inflammatory activity.

Some authors suggested that the significance of humoral CSF changes could change from a mere diagnostic finding to a valuable therapeutic biomarker that could help assess the effective control of CNS-established inflammation in MS [41]. However, in individual cases, longitudinal changes in concentration would need to be investigated using serum/CSF samples in order to use FLCκ as a marker of therapeutic response. It should be kept in mind that the use of a CSF-based therapeutic biomarker is always inferior to blood-based biomarkers due to the invasive procedure of lumbar puncture.

### Limitations

A major limitation of this study is the lack of longitudinal analyses of FLCκ concentrations before and after DMT intake. Therefore, a direct effect of DMT on FLCκ concentrations cannot be proven in individual cases. On the other hand, due to the cross-sectional design of the study, we were able to detect a statistically significant effect that could indicate a relationship between DMT intake and FLCκ concentrations. Further longitudinal analyses are needed to confirm our findings. Another limitation is the small number of patient samples per DMT cohort, especially in the cohort of highly effective DMT and the analysis of drugs classified by clinical efficacy rather than comparing individual drugs. The retrospective design and the bias due to diagnostic CSF analysis performed in some cases to exclude PML are also limitations of our study. Since this is a retrospective data analysis, information on flow cytometric analyses of the B cell repertoire is not included.

## 5. Conclusions

Very highly effective DMT is associated with reduced intrathecal concentrations of IgG and FLCκ. The reduction in FLCκ could contribute to an anti-inflammatory effect in the CNS via this mechanism. Longitudinal analyses are needed to confirm these findings.

## Figures and Tables

**Table 1:** Clinical and CSF data.

	DMT Moderate Efficacy (*n* = 20)	DMT High Efficacy (*n* = 10)	DMT Very High Efficacy (*n* = 30)	Patients without DMT (*n* = 90)	*p*-Value
age	49 (44;56)	38 (30;42)	37 (32;48)	38 (27;48)	0.004
female (*n*; %)	12 (60)	5 (50)	27 (90)	60 (67)	0.018
number of DMT before actual therapy	0 (0;1)	2 (1;3)	2 (1;3)	-	
DMT	INFß-1a *n* = 5	fingolimod *n* = 7	alemtuzumab *n* = 8		
	INFß-1b *n* = 3	daclizumab *n* = 3	natalizumab *n* = 14		
	glatiramer acetate *n* = 2		rituximab/ocrelizumab *n* = 7		
	dimethyl fumarate *n* = 4		mitoxantrone *n* = 1		
	teriflunomide *n* = 3				
	triamcinolone *n* = 3				
Mean duration of intake	3.47 years	344 days			
Interval between last drug administration and LP in days			alemtuzumab 359 (147;468) anti-CD20 therapies 88 (39;143) natalizumab 30 (14;40)		
Disease duration (y)	9.5 (4;18)	12 (7;13)	7 (4;10.75)		
EDSS	3 (1.5;5)	5.8 (3;6.9)	5 (2.6;6.4)	1.8 (1;2.6)	<0.0001
MRI Gd enhancement (*n*; %)	2/16 (12.5)	4/8 (50)	5/26 (19.2)	53/87 (60.9)	<0.001
ARR * before LP	0.3	0.7	0.8	-	0.08
NEDA-3 (*n*; %) before LP	8/18 (44.4)	0/9	5/30 (16.7)	-	0.017
MS type	RRMS *n* = 15	RRMS *n* = 9	RRMS *n* = 24	RRMS *n* = 52	
	PMS *n* = 5	PMS *n* = 1	PMS *n* = 6	CIS *n* = 30, PMS *n* = 8	
CSF data					
Qalb * 10^−3^	6.8 (4.41;9.89)	6.1 (5.4;6.9)	5.1 (4;7.1)	5.1 (4.2;6.8)	0.349
QigG * 10^−3^	4.6 (3.55;6.83)	6.2 (5.4;9)	4 (3;6.4)	4.4 (3.1;5.9)	0.129
IgG loc	52.3 (41.5;59)	62.7 (48.3;74.2)	34 (23;48)	46 (34;69.9)	0.005
IgG IF	0 (0;21.5)	21 (16;47)	0 (0;31)	5 (0;38)	0.264
OCB pos (*n*; %)	19/20 (95)	9/9 (100)	26/30 (87)	85/90 (94)	0.826
FLCκ serum (mg/L)	11.9 (9.3;14.5)	10.6 (9.2;12.9)	10.7 (7.5;11.8)	11.8 (9.3;14)	0.067
FLCκ CSF (mg/L)	2.3 (0.95;4.7)	6 (2.6;9.98)	1.2 (0.4;2.3)	3.7 (1.1; 6.6)	<0.0001
FLCκ index	25.8 (18.9;77.1)	96.4 (56.6;186.9)	26.5 (6.98;56.2)	58.6 (18.4;123.4)	0.002
FLCκ loc	2.2 (0.75;4.53)	5.9 (2.4;9.9)	1.1 (0.3;2.1)	3.49 (0.96;6.4)	<0.0001
FLCκ IF	88 (80;95)	97 (93;98)	88 (55;92)	94 (81;97)	0.001

Continuous data are displayed as median (1st; 3rd quartile); nominal data are given as percentages. EDSS—expanded disability status scale, CSF—cerebrospinal fluid, Qalb—albumin quotient, QIgG—immunoglobulin G quotient, OCB—oligoclonal band, FLCκ—free light chains kappa, IF—intrathecal fraction, MS—multiple sclerosis, DMT—disease-modifying therapy, LP—lumbar puncture, RRMS—relapsing–remitting MS, PMS—progressive MS, INF—interferon, Gd—gadolinium, NEDA—no evidence of disease activity [25], ARR—annualized relapse rate; * defined as the total number of relapses per patient per year. Intergroup comparison was performed using the Kruskal–Wallis test or the Fisher–Freeman–Halton exact test for nominal data. *p* values ≤ 0.05 were regarded as statistically significant.

## Data Availability

The data that supports the findings of this study are available on request from the corresponding author. The data are not publicly available due to privacy or ethical restrictions.
